# The Generalized Relative Pairs IBD Distribution: Its Use in the Detection of Linkage

**DOI:** 10.3389/fpubh.2016.00259

**Published:** 2016-11-23

**Authors:** Quan Zou

**Affiliations:** ^1^Department of Statistics, The George Washington University, Washington, DC, USA

**Keywords:** allele identical by descent, *ITO* method, quantitative trait locus, relative pairs, linkage analysis

## Abstract

I introduce a novel approach to derive the distribution of disease affectional status given alleles *identical by descent* (*IBD*) sharing through *ITO* method. My approach tremendously simplifies the calculation of the affectional status distribution compared to the conventional method, which requires the parental mating information, and could be applied to disease with both dichotomous trait and *quantitative trait locus* (*QTL*). This distribution is shown to be independent of relative relationship and be employed to develop the marker *IBD* distributions for relative relationship. In addition, three linkage tests: the proportion, the mean test, and the LOD score test are proposed for different relative pairs based on their marker *IBD* distributions. Among all three tests, the mean test for sib pair requires the least sample size, thus, has the highest power. Finally, I evaluate the significance of different relative relationships by a Monte-Carlo simulation approach.

## 1 Introduction

Upon the completion of human genome sequences, genetic markers have enabled mapping of human disease genes through linkage analysis. Sib pairs are the most common design among all possible family configurations. A variety of linkage analyses have been developed for testing *identical by descent* (*IBD*) sharing of affected sib pairs. Penrose first considered the covariance of the quantitative sib pair trait phenotype and genetic marker in the linkage analysis (1). Haseman and Elston logistically regressed the squared quantitative trait difference on the shared alleles *IBD* in sib pairs (2). Suarez illustrated the perturbations in the marker *IBD* for sib pair to detect linked dichotomous trait locus (3). Risch applied recurrence risk ratio method to investigate the *IBD* sharing of affected sib pairs with dichotomous traits and has also extended this method to other relative pairs (4, 5). Amos showed that a variance components procedure could assess the genetic linkage (6, 7). The model also accommodates gene–environment interactions and the effects of covariates and epistasis.

The basic principle of linkage analysis is the similarity between disease trait and marker genotype, which are measured by (disease) affectional status and (marker) alleles *IBD* of the relative pairs, respectively. If the trait and marker loci are linked, relative pair, that is likely to share disease alleles, is also likely to inherit the same marker allele or *vice versa*. Thus, doubly affected sib pair should show greater than expected chance of sharing two linked maker alleles *IBD*. Using the similarity measure of geno- and phenotype, several statistical tests for linkage can be constructed by deriving the expected degree of similarity under certain linkage assumption. The simplest approach is chi-square “goodness of fit” test to compare the observed and expected marker alleles *IBD* under the hypothesis of no linkage (8). The proportion test based on the counts of doubly affected sib pairs with two marker alleles *IBD*, was proposed by Day and Simons and Suarez et al. (3, 9). The mean test, suggested by de Vries et al. and Green and Woodrow, is based on the average number of marker alleles *IBD* weighted by their probabilities (10, 11). The mean test is generally more powerful than the proportion and the goodness-of-fit tests (12). Another type of method is likelihood ratio test, which utilizes LOD score of the proportion of marker alleles *IBD* (4). The power of likelihood ratio test can be increased by restricting *IBD* proportions to certain genetic models (13, 14).

The *ITO* method refers to the stochastic matrices developed by Li and Sacks, where *I, T*, and *O* denote the probabilities sharing 2, 1, and 0 alleles *IBD* given relative pairs’ genotypes, respectively (15). These *ITO* matrices provide a simple relationship between relative genotypes and their *IBD* status and have been wildly used in genetic analysis (16, 17). For example, the conditional genotype probabilities of sib pairs could be calculated from *ITO* matrices (18). The general formulation of genotype distributions of other relative pairs are also suggested by using the *ITO* method (19). The ordered *ITO* transition matrices were extended to calculate the genetic covariance (20).

In order to examine the *IBD* sharing within affected family, Risch has shown that the *IBD* probabilities of affected relative pair depend on the recurrence risk ratio, known as *λ* (4). Under the assumption of incompletely penetrant model, the probabilities of the sibling’s affectional status given alleles *IBD* sharing could also be recovered from Table II in Haseman and Elston, by conditioning on parental mating types (2, 3). However, this approach will require the information of second-degree parental mating when being applied to relative relationships other than sib pair. In the present paper, I partition the relative pairs’ affectional status on their genotype information with respect to alleles *IBD* sharing, *i.e*., the *ITO* matrices. The *ITO* method greatly simplifies the derivation of the conditional distribution of affectional status for both the quantitative and the dichotomous traits. Furthermore, it is shown that these probabilities are independent of relative relationships.

In this research, I adopt a novel *ITO* method and develop the allelic *identical by descent* (*IBD*) distributions at marker locus given disease affectional status for siblings, uncle–nephew, grandparent–grandchild, half sibs, and first cousin pairs. By taking advantage of the *ITO* matrices, I first demonstrate that the probabilities of dichotomous disease status given trait *IBD* score are independent of relative relationships. Then, I fully derive the marker *IBD* distributions given dichotomous disease affectional status for various relative relationships by utilizing the relative pairs’ joint probabilities of *IBD* scores at both trait and marker loci. I also calculate the marker *IBD* distributions given extreme discordant relative pairs at a *quantitative trait locus* (*QTL*) for different relative relationships by my novel *ITO* method. Next, I examine the power to detect the presence of a significant disease susceptibility locus through linkage analysis by perturbing the conditional marker *IBD* distribution. Specifically, three tests, the proportion test, the mean test, and the logarithm of odds (LOD) score test, were applied to obtain the sample size required to achieve significance level *p* with different power. Finally, the Monte-Carlo simulation studies have been conducted in order to evaluate the performance of my methods. I assume Hardy–Weinberg equilibrium, random mating and the marker locus to be completely polymorphic such that all matings are informative.

## 2 Materials and Methods

Let us consider the situation where alleles (*T*/*t*) at the trait locus are linked to alleles (*M*/*m*) at a marker locus through recombination fraction *θ* and assume that the marker locus is completely polymorphic. Additionally, the diallelic frequencies are *p* and *q* for alleles *T* and *t*, where *p* + *q* = 1. I denote penetrance frequencies, *i.e*., the probability of the affected relative given genotypes *TT, Tt*, or *tt* by *f*_1_, *f*_2_, or *f*_3_, respectively. The prevalence of the trait in the population is defined as *K_P_* = *p*^2^*f*_1_ + 2*pq f*_2_ + *q*^2^*f*_3_, in addition to the additive variance (*V_A_* = 2*pq*[*p*(*f*_2_ − *f*_1_) + *q*(*f*_3_ − *f*_2_)]^2^) and dominance variance (*V_D_* = *p*^2^*q*^2^(*f*_1_ − 2*f*_2_ + *f*_3_)^2^). I assume no major gene by residual interaction and no epistasis, *i.e*., the non-allelic interaction of different genes.

### 2.1 The Conditional Marker *IBD* Given the Affected Status Distributions

Let *X* denotes the number of affected individuals in a relative pair. In order to calculate the conditional probabilities of *X* = *k* (*k* = 0, 1, 2) given *IBD* score at trait locus for generalized relative pairs, I reckon the genotype information of relative pairs derived from the *ITO* matrices, as shown in Table 1 (15).

**Table 1:** **The conditional distributions of relative pair with genotypes (*G*_1_–*G*_2_) given the trait *IBD* values**.

		*IBD_T_* = 2 (I)			*IBD_T_* = 1 (T)			*IBD_T_* = 0 (O)	
*G*_2_		*T T*	*Tt*	*tt*	*T T*	*Tt*	*tt*	*T T*	*Tt*	*tt*
	*T T*	*p*^2^			*p*^3^	*p*^2^*q*		*p*^4^	2*p*^3^*q*	*p*^2^*q*^2^
*G*_1_	*Tt*		2*pq*		*p*^2^*q*	*pq*	*pq*^2^	2*p*^3^*q*	4*p*^2^*q*^2^	2*pq*^3^
	*tt*			*q*^2^		*pq*^2^	*q*^3^	*p*^2^*q*^2^	2*pq*^3^	*q*^4^

The conditional affected status given *IBD_T_* (*t* = 0, 1, 2) probabilities has been partitioned on all possible genotypes of relative pairs, *GT_i_, i* = 1, 2, …, 9: *TT − TT, TT − Tt, TT − tt, Tt − TT, Tt − Tt, Tt − tt, tt − TT, tt − Tt*, and *tt − tt*, as shown in equation (1):

(1) $$\begin{array}{l} \mathrm{Pr}\left(X=k|\mathrm{IB}D_{T}=t\right)\\ =\sum_{i=1}^{9}\mathrm{Pr}\left(X=k,GT_{i}|\mathrm{IB}D_{T}=t\right)\\ =\sum_{i=1}^{9}\mathrm{Pr}\left(X=k|GT_{i}\right)\cdot \mathrm{Pr}\left(GT_{i}|\mathrm{IB}D_{T}=t\right). \end{array}$$

Note that I have utilized the fact that the affected status of relative pair is conditionally independent of trait *IBD* score, given their genotype. Clearly, knowledge of the trait *IBD* score provides no extra information on the likelihood of affected status given their genotype. For example, given *IBD_T_* = 2, there are only 3 genotypes of the relative pair involved: *TT − TT, Tt − Tt*, and *tt − tt*, which implies that

(2) $$\begin{array}{l} \mathrm{Pr}\left(X=2|\mathrm{IB}D_{T}=2\right)\\ =\sum_{i=1}^{9}\mathrm{Pr}\left(X=2|GT_{i}\right)\cdot \mathrm{Pr}\left(GT_{i}|\mathrm{IB}D_{T}=2\right)\\ =f_{1}^{\;2}p^{2}+2f_{2}^{\;2}\mathrm{pq}+f_{3}^{\;2}q^{2}\\ =K_{P}^{2}+V_{A}+V_{D}. \end{array}$$

The resulting *Pr*(*X* = *k* | *IBD_T_* = *t*) as in Table 1 of Suarez was reproduced here in Table 2 by Li’s *ITO* method (3, 15). Throughout the calculation, I merely depend on the *ITO* matrices and trait genotype penetrance frequencies *f*_1_, *f*_2_, and *f*_3_. It is easy to see that conditional distribution of affected status on *IBD* score at trait locus is independent of relative relationships. Indeed, the affected number of relative pair should only depend on the numbers of trait alleles shared between the relative pairs.

**Table 2:** **The conditional distributions of the affected status given the trait *IBD* values**.

	Pr(*X =* *k|IBD_T_ =* *t*)		
No. of affectedpairs	*t* = 2	*t* = 1	*t* = 0
*X* = 2	$K_{P}^{2}+V_{A}+V_{D}$	$K_{P}^{2}+\frac{V_{A}}{2}$	$K_{P}^{2}$
*X* = 1	$2\left(K_{P}-K_{P}^{2}-V_{A}-V_{D}\right)$	$2K_{P}-2K_{P}^{2}-V_{A}$	$2K_{P}-2K_{P}^{2}$
*X* = 0	$2-2K_{P}+K_{P}^{2}+V_{A}+V_{D}$	$2-2K_{P}+K_{P}^{2}+\frac{V_{A}}{2}$	$2-2K_{P}+K_{P}^{2}$

The probabilities of *IBD* at trait locus, *Pr*(*IBD_T_* = *t*) (*t* = 0, 1, 2), for sib pair, grandparent–grandchild, uncle–nephew, half sib, and first cousin are given in Table 3. By Bayes’ theorem, *Pr*(*X*) = Σ*_t_* *Pr*(*X |* *IBD_T_* = *t*)⋅*Pr*(*IBD_T_* = *t*), I give the marginal affected status probabilities for different relative relationships from a randomly mating population in Table 4.

**Table 3:** **The *IBD* probabilities at the trait locus**.

	*IBD_T_ = t*		
Relationship	*t* = 2	*t* = 1	*t* = 0
Sibs	$\frac{2}{2}$	$\frac{2}{2}$	$\frac{2}{2}$
Grandparent–grandchild			
Uncle–nephew	0	$\frac{2}{2}$	$\frac{2}{2}$
Half sibs			
First cousins	0	$\frac{2}{2}$	$\frac{2}{2}$

**Table 4:** **The marginal distributions of the affected status**.

	Affected relative pairs (*X =* *k*)		
Relationship	*X* = 2	*X* = 1	*X* = 0
Sibs (3)	$K_{P}^{2}+\frac{V_{A}}{2}+\frac{V_{D}}{2}$	$2K_{P}-2K_{P}^{2}$	$2-2K_{P}+K_{P}^{2}$
		$-V_{A}-\frac{V_{D}}{2}$	$+\frac{V_{A}}{2}+\frac{V_{D}}{2}$
Grandparent– grandchild			
Uncle–nephew	$K_{P}^{2}+\frac{V_{A}}{2}$	$2K_{P}-2K_{P}^{2}-\frac{V_{A}}{2}$	$2-2K_{P}+K_{P}^{2}+\frac{V_{A}}{2}$
Half sibs			
First cousins	$K_{P}^{2}+\frac{V_{A}}{2}$	$2K_{P}-2K_{P}^{2}-\frac{V_{A}}{2}$	$2-2K_{P}+K_{P}^{2}+\frac{V_{A}}{2}$

Let the *IBD* scores at the marker and trait loci be denoted by *IBD_M_* and *IBD_T_*, respectively. The joint probabilities for a relative pair to have *IBD* scores at the marker locus *M* and the number of affected relative pair is calculated as equation (3).

(3) $$\begin{array}{l} \mathrm{Pr}\left(\mathrm{IB}D_{M}=m,X=k,r\right)\\ =\sum_{t}^{}\mathrm{Pr}\left(X=k|\mathrm{IB}D_{T}=t\right)\cdot \mathrm{Pr}\left(\mathrm{IB}D_{M}=m,\mathrm{IB}D_{T}=t,r\right). \end{array}$$

where relationship subscript *r* (relationship) refers *s* (sib), *g* (grandparent–grandchild), *u* (uncle–nephew), *h* (half sib), and *f* (first cousin). One notices that conditional probabilities *Pr*(*X* = *x*|*IBD_T_* = *t*) are independent of the relative relationships. Hence, the differences among relative relationships of the joint probabilities *Pr*(*IBD_M_* = *m, X* = *k, r*) are due to the contribution of *Pr*(*IBD_M_* = *m, IBD_T_* = *t,r*). Combining *Pr*(*IBD_M_* = *m, IBD_T_* = *t*) [see Table 1 in Risch (5)] and Table 4 according to equation (3), I obtain Table 5, in which *θ* is the recombination fraction between the trait and marker loci, parameter *ψ* defines *θ*^2^ + (1 − *θ*)^2^.

**Table 5:** **The conditional marker *IBD* given the affected status distribution**.

	Affected relative pairs (*X =* *k*)		
Relationship and *IBD_M_ = m*	*X* = 2	*X* = 1	*X* = 0
**Sibs (3)**
*m* = 2	$\frac{1}{4}+\frac{\left(\psi -\frac{1}{2}\right)V_{A}+\left(\psi^{2}-\frac{1}{4}\right)V_{D}}{4\left(K_{P}^{2}+\frac{V_{A}}{2}+\frac{V_{D}}{4}\right)}$	$\frac{1}{4}-\frac{\left(\psi -\frac{1}{2}\right)V_{A}+\left(\psi^{2}-\frac{1}{4}\right)V_{D}}{2\left(2K_{P}-2K_{P}^{\;2}-V_{A}-\frac{V_{D}}{2}\right)}$	$\frac{1}{4}+\frac{\left(\psi -\frac{1}{2}\right)V_{A}+\left(\psi^{2}-\frac{1}{4}\right)V_{D}}{4\left(1-2K_{P}+K_{P}^{\;2}+\frac{V_{A}}{2}+\frac{V_{D}}{4}\right)}$
*m* = 1	$\frac{1}{2}-\frac{{\left(\psi -\frac{1}{2}\right)}^{2}V_{D}}{2\left(K_{P}^{\;2}+\frac{V_{A}}{2}+\frac{V_{D}}{2}\right)}$	$\frac{1}{2}+\frac{{\left(\psi -\frac{1}{2}\right)}^{2}V_{D}}{2K_{P}-2K_{P}^{\;2}-V_{A}-\frac{V_{D}}{2}}$	$\frac{1}{2}-\frac{{\left(\psi -\frac{1}{2}\right)}^{2}V_{D}}{2\left(1-2K_{P}+K_{P}^{\;2}+\frac{V_{A}}{2}+\frac{V_{D}}{4}\right)}$
*m* = 0	$\frac{1}{4}-\frac{\left(\psi -\frac{1}{2}\right)V_{A}+\left(2\psi -\psi^{2}-\frac{3}{4}\right)V_{D}}{4\left(K_{P}^{\;2}+\frac{V_{A}}{2}+\frac{V_{D}}{4}\right)}$	$\frac{1}{4}+\frac{\left(\psi -\frac{1}{2}\right)V_{A}+\left(2\psi -\psi^{2}-\frac{3}{4}\right)V_{D}}{2\left(2K_{P}-2K_{P}^{\;2}-V_{A}-\frac{V_{D}}{2}\right)}$	$\frac{1}{4}-\frac{\left(\psi -\frac{1}{2}\right)V_{A}+\left(2\psi -\psi^{2}-\frac{3}{4}\right)V_{D}}{4\left(1-2K_{P}+K_{P}^{\;2}+\frac{V_{A}}{2}+\frac{V_{D}}{4}\right)}$
**Grandparent–grandchild**			
*m* = 1	$\frac{1}{2}+\frac{\left(\frac{1}{2}-\theta \right)V_{A}}{4\left(K_{P}^{\;2}+\frac{V_{A}}{4}\right)}$	$\frac{1}{2}-\frac{\left(\frac{1}{2}-\theta \right)V_{A}}{2\left(2K_{P}-2K_{P}^{\;2}-\frac{V_{A}}{2}\right)}$	$\frac{1}{2}+\frac{\left(\frac{1}{2}-\theta \right)V_{A}}{4\left(1-2K_{P}+K_{P}^{\;2}+\frac{V_{A}}{4}\right)}$
*m* = 0	$\frac{1}{2}-\frac{\left(\frac{1}{2}-\theta \right)V_{A}}{4\left(K_{P}^{\;2}+\frac{V_{A}}{4}\right)}$	$\frac{1}{2}+\frac{\left(\frac{1}{2}-\theta \right)V_{A}}{2\left(2K_{P}-2K_{P}^{\;2}-\frac{V_{A}}{2}\right)}$	$\frac{1}{2}-\frac{\left(\frac{1}{2}-\theta \right)V_{A}}{4\left(1-2K_{P}+K_{P}^{\;2}+\frac{V_{A}}{4}\right)}$
**Uncle–nephew**
*m* = 1	$\frac{1}{2}+\frac{\left[\psi \left(2-\theta \right)+\frac{\theta }{2}-\frac{1}{2}\right]V_{A}}{4\left(K_{P}^{\;2}+\frac{V_{A}}{4}\right)}$	$\frac{1}{2}-\frac{\left[\psi \left(1-\theta \right)+\frac{\theta }{2}-\frac{1}{2}\right]V_{A}}{2\left(2K_{P}-2K_{P}^{\;2}-\frac{V_{A}}{2}\right)}$	$\frac{1}{2}+\frac{\left[\psi \left(1-\theta \right)+\frac{\theta }{2}-\frac{1}{2}\right]V_{A}}{4\left(1-2K_{P}+K_{P}^{\;2}+\frac{V_{A}}{4}\right)}$
*m* = 0	$\frac{1}{2}-\frac{\left[\psi \left(1-\theta \right)+\frac{\theta }{2}-\frac{1}{2}\right]V_{A}}{4\left(K_{P}^{\;2}+\frac{V_{A}}{4}\right)}$	$\frac{1}{2}+\frac{\left[\psi \left(1-\theta \right)+\frac{\theta }{2}-\frac{1}{2}\right]V_{A}}{2\left(2K_{P}-2K_{P}^{\;2}-\frac{V_{A}}{2}\right)}$	$\frac{1}{2}-\frac{\left[\psi \left(1-\theta \right)+\frac{\theta }{2}-\frac{1}{2}\right]V_{A}}{4\left(1-2K_{P}+K_{P}^{\;2}+\frac{V_{A}}{4}\right)}$
**Half-sibs**
*m* = 1	$\frac{1}{2}+\frac{\left(\psi -\frac{1}{2}\right)V_{A}}{4\left(K_{P}^{\;2}+\frac{V_{A}}{4}\right)}$	$\frac{1}{2}-\frac{\left(\psi -\frac{1}{2}\right)V_{A}}{2\left(2K_{P}-2K_{P}^{\;2}-\frac{V_{A}}{2}\right)}$	$\frac{1}{2}+\frac{\left(\psi -\frac{1}{2}\right)V_{A}}{4\left(1-2K_{P}+K_{P}^{\;2}+\frac{V_{A}}{4}\right)}$
*m* = 0	$\frac{1}{2}-\frac{\left(\psi -\frac{1}{2}\right)V_{A}}{4\left(K_{P}^{\;2}+\frac{V_{A}}{4}\right)}$	$\frac{1}{2}+\frac{\left(\psi -\frac{1}{2}\right)V_{A}}{2\left(2K_{P}-2K_{P}^{\;2}-\frac{V_{A}}{2}\right)}$	$\frac{1}{2}-\frac{\left(\psi -\frac{1}{2}\right)V_{A}}{4\left(1-2K_{P}+K_{P}^{\;2}+\frac{V_{A}}{4}\right)}$
**First cousins**
*m* = 1	$\frac{1}{4}+\frac{\left[(\psi {\left(1-\theta \right)}^{2}+\frac{1}{2}\theta^{2}-\frac{1}{4}\right]V_{A}}{8\left(K_{P}^{\;2}+\frac{V_{A}}{8}\right)}$	$\frac{1}{4}-\frac{\left[(\psi {\left(1-\theta \right)}^{2}+\frac{1}{2}\theta^{2}-\frac{1}{4}\right]V_{A}}{4\left(2K_{P}-2K_{P}^{\;2}-\frac{V_{A}}{4}\right)}$	$\frac{1}{4}+\frac{\left[(\psi {\left(1-\theta \right)}^{2}+\frac{1}{2}\theta^{2}-\frac{1}{4}\right]V_{A}}{8\left(1-2K_{P}+K_{P}^{\;2}+\frac{V_{A}}{8}\right)}$
*m* = 0	$\frac{3}{4}-\frac{\left[(\psi {\left(1-\theta \right)}^{2}+\frac{1}{2}\theta^{2}-\frac{1}{4}\right]V_{A}}{8\left(K_{P}^{\;2}+\frac{V_{A}}{8}\right)}$	$\frac{3}{4}+\frac{\left[(\psi {\left(1-\theta \right)}^{2}+\frac{1}{2}\theta^{2}-\frac{1}{4}\right]V_{A}}{2\left(2K_{P}-2K_{P}^{\;2}-\frac{V_{A}}{4}\right)}$	$\frac{3}{4}-\frac{\left[(\psi {\left(1-\theta \right)}^{2}+\frac{1}{2}\theta^{2}-\frac{1}{4}\right]V_{A}}{8\left(1-2K_{P}+K_{P}^{\;2}+\frac{V_{A}}{8}\right)}$

### 2.2 Extreme Discordant Relative Pair for Quantitative Trait Locus (QTL)

Risch and Zhang have shown that sib pairs from opposite tails of the phenotypic distribution have substantial power to detect linkage for a quantitative trait locus (*QTL*) (21, 22). Assuming the Haseman and Elston model, *x* denotes the individual observed phenotypic value: *x* = *μ* + *g* + *e*, where *μ* is the general mean, *g* and *e* are the genetic and environmental effects, respectively (2). Following Risch and Zhang, define biallelic locus (*T*/*t*) with gene frequencies *p* and *q*, respectively (21). Let *a* be the mean value of genetic effect being *TT, d* the mean being *Tt*, and −*a* being *tt*. Without loss of generality, I assume *a* = 1, *d* = 0, residual variance within each genotype $\sigma_{e}^{2}=1$ and no residual correlation between relative pairs, *i.e*., *ρ* = 0. Therefore, the cumulative distribution function *F*(*x*) for the population distribution of the trait is a mixture of three normal distributions:

(4) $$F\left(x\right)=\int_{-\infty }^{x}\left[\;p^{2}\phi \left(s-1\right)+2\mathrm{pq}\phi \left(s\right)+q^{2}\phi \left(s+1\right)\right]\mathrm{ds}.$$

where *ϕ*(*s*) is the standard normal density function. Next, the probability of one relative’s phenotype falls in the top decile and the other relative’s in the bottom decile given their trait genotypes, *Pr*(*T*_1_*B*_1_|*GT_i_*) (*i* = 1, 2, …, 9), is given as

(5) $$\mathrm{Pr}\left(T_{1}B_{1}|GT_{i}\right)=\int_{-\infty }^{F^{-1}\left(0.1\right)}\int_{F^{-1}\left(0.9\right)}^{\infty }\phi \left(s,t:G_{1},G_{2}\right)\mathrm{dsdt},$$

where *ϕ*(*s, t*) is the bivariate normal density function, *G*_1_, *G*_2_ take 1, 0, or −1 as their genotypes are *TT, Tt*, or *tt*, respectively. Thus, the probabilities of the general extreme discordant relative pair given allele *IBD* sharing at trait locus is obtained through *ITO* method:

(6) $$\begin{array}{l} \mathrm{Pr}\left(T_{1}B_{1}|\mathrm{IB}D_{T}=t\right)\\ =\sum_{i=1}^{9}\mathrm{Pr}\left(T_{1}B_{1},GT_{i}|\mathrm{IB}D_{T}=t\right)\\ =\sum_{i=1}^{9}\mathrm{Pr}\left(T_{1}B_{1}|GT_{i}\right)\cdot \mathrm{Pr}\left(GT_{i}|\mathrm{IB}D_{T}=t\right), \end{array}$$

where *Pr*(*T*_1_*B*_1_|*GT_i_*) is integrated according to equation (5), and *Pr*(*GT_i_*|*IBD_T_* = *t*) are the *ITO* matrices given in Table 1. Again, the probabilities of extreme discordant relative pair with *QTL* given *IBD_T_* are partitioned over their genotypes through the *ITO* approach. Similar to the discrete case, *Pr*(*T*_1_*B*_1_|*IBD_T_* = *t*) is also independent to the relative relationships. If one regards the extreme discordant relative pair with *QTL* as the continuous case for *X* = 1, then the probabilities of *Pr*(*T*_1_*B*_1_) and *Pr*(*IBD_M_*|*T*_1_*B*_1_) could be derived in a similar fashion as in the discrete case.

## 3 Results

The power to detect linkage will naturally decrease as the distance between the trait (*T*/*t*) and marker (*M*/*m*) loci decreases. Here, I refer the perturbation as the absolute deviation of the conditional probabilities in Table 5 from those under the null hypothesis, *i.e*., $|\mathrm{Pr}{\left(\mathrm{IB}D_{M}|X\right)}_{\theta }-\mathrm{Pr}{\left(\mathrm{IB}D_{M}|X\right)}_{\theta_{0}}|$. In general, the less perturbation is, the harder the linkage is detected. I fix a reasonable *K_P_* value as 10% and focus on doubly affected relative pairs. In order to compare the test power between the full sib pair and other relative relationships, I let *V_D_* = 0.01 such that the perturbation of sib pairs is increasing as *V_A_* increases (3). For extreme discordant relative pairs with *QTL*, I use an additive model with *p* = 0.8, *a* = 1, *d* = 0, $\sigma_{e}^{2}=1$, and *ρ* = 0. High recessive frequency allele with correlated residual will yields the maximal perturbation in the conditional marker *IBD* probabilities, *i.e*., the perturbation increases, as allele frequency *p* or phenotype value of heterozygote *d* decreases, or residual correlation *ρ* increases (21, 22). In this section, I derive both common Wald- and score-type tests with either binary or continuous trait. Further, I consider the Monte-Carlo simulation to validate the power of the previous tests.

### 3.1 Proportion Test

I define *N_j_* (*j* = 2 for sib pair and *j* = 1 for other relative pairs) as the counts of doubly affected relative pairs with the dichotomous trait or extreme discordant relative pairs with *QTL*, which share *j* marker allele(s) *IBD* among total *N* relative pairs sampled. The Wald test statistic is

(7) $$W_{r}=\frac{N_{j}-E\left(N_{j}\right)}{\sqrt{\text{Var}\left(N_{j}\right)}}.$$

Under the alternative hypothesis that $\theta <\frac{1}{2}$, *N_j_* is approximately normally distributed with

(8) $$\begin{array}{rr} N\left(\frac{\sqrt{N_{r}}\left(4\epsilon_{r}-1\right)}{\sqrt{3}},\frac{16\epsilon_{r}\left(1-\epsilon_{r}\right)}{3}\right)\;, & \text{for}\;r=s,f;\\ N\left(\sqrt{N_{r}}\left(2\epsilon_{r}-1\right),4\epsilon_{r}\left(1-\epsilon_{r}\right)\right)\;, & \text{for}\;r=g,u,h. \end{array}$$

by Central Limit Theorem, where $\epsilon_{r}$ refers to conditional marker *IBD* probabilities of relative relationship found in Table 5.

Since all the *IBD* perturbations are monotonic based on the parameters chosen, the proportion tests are one-sided: *W_r_* > *Z_*α*_* for doubly affected relative pairs and *W_r_* < −*Z_*α*_* for relative pairs with *QTL*. The required sample size *N_r_* for this test to have the power of 1 − *β* is (14):

(9) $$\begin{array}{ll} N_{r}={\left(\frac{\pm \;\sqrt{3}Z_{\alpha }-4Z_{1-\beta }\sqrt{\epsilon_{r}\left(1-\epsilon_{r}\right)}}{4\epsilon_{r}-1}\right)}^{2}, & \text{for}\;r=s,f;\\ N_{r}={\left(\frac{\pm \;Z_{\alpha }-2Z_{1-\beta }\sqrt{\epsilon_{r}\left(1-\epsilon_{r}\right)}}{2\epsilon_{r}-1}\right)}^{2}, & \text{for}\;r=g,u,h. \end{array}$$

**Figure 1:**
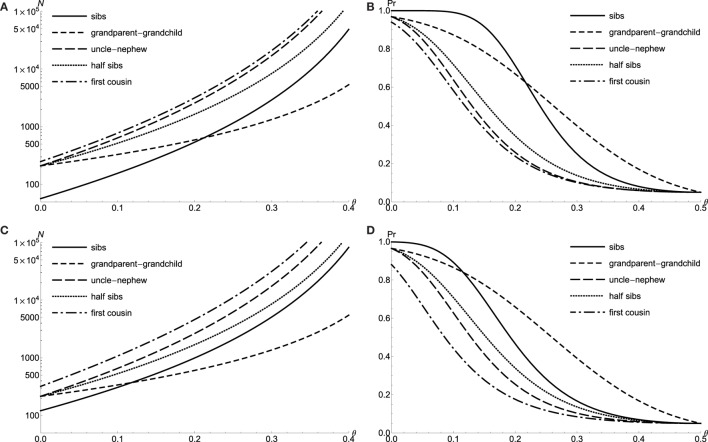
**The proportion test power for doubly affected relative pairs with the dichotomous trait and extreme discordant relative pairs with *QTL***. Required sample size *N* of level *α* = 0.05 proportion test with 90% power to detect linkage *θ* for doubly affected relative pairs **(A)** and extreme discordant relative pairs with *QTL* **(C**). Power to detect linkage *θ* of level *α* = 0.05 proportion test by using *N* = 300 doubly affected relative pairs **(B)** and *N* = 300 extreme discordant relative pairs with *QTL* **(D)**.

As previously noted, I take the parameters of *K_P_* = 0.1 and *V_A_* = 0.01 for doubly affected relative pair with the dichotomous trait, and consider the level *α* = 0.05 proportion test with 90% power to detect the linkage for various relative types. Figure 1A shows that the required sample size *N* plotted as a function of recombination fraction *θ*. The power is calculated for a sample of *N* = 300 relative pairs (Figure 1B). The power of test for sib pair (solid line) is uniformly larger than that of first cousin (dotted dash), which is explained by larger marker *IBD* perturbation of sib pairs. However, grandparent–grandchild has the best power among all five relative relationships, when *θ* > 0.217 (Figure 1B). The increasing test power of grandparent–grandchild relative pair is due to the less decrease in perturbation when *θ* is large. The grandparent–grandchild relative pair dominates the test power among other relative relationships whenever $\theta \geq \frac{1}{4}$, which is consistent with the results of Risch (4). For extreme discordant relative pairs with *QTL*, the results are similar to the case of doubly affected relative pairs with the dichotomous trait (see Figures 1C,D).

### 3.2 LOD Score Test

Following previous notation, the kernel of the likelihood of *N_j_* (*j* = 2 for sib pair, *j* = 1 for other relatives) is the following:

(10) $${\left(\frac{N_{j}}{N}\right)}^{N_{j}}{\left(\frac{N-N_{j}}{N}\right)}^{N-N_{j}}.$$

Note that the parameter of interest is not the recombination fraction *θ* any more, but *N_j_*, the count of relative pairs sharing *j* allele(s) *IBD*. With $\hat{\epsilon }=\frac{N_{j}}{N}$ denoting the ML estimates for ϵ as it varies in the parameter space, then the LOD score *T* for the likelihood ratio test based on equation (10) is given by

**Figure 2:**
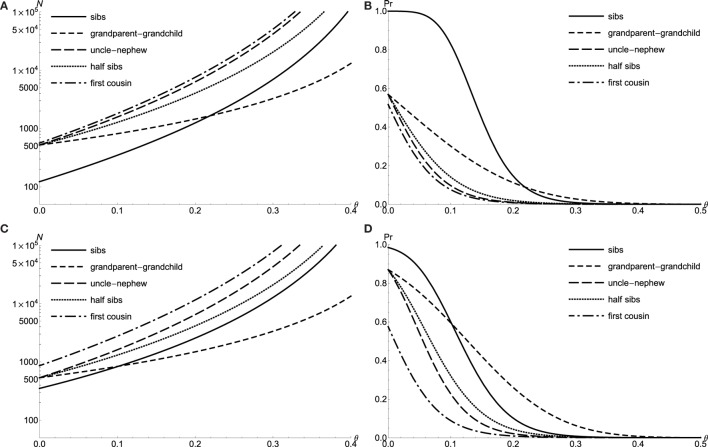
**The LOD test power for doubly affected relative pairs with the dichotomous trait and extreme discordant relative pairs with *QTL***. Required sample size *N* of level *α* = 0.001 LOD score test with 90% power to detect linkage *θ* for doubly affected relative pairs **(A)** and extreme discordant relative pairs with *QTL* **(C)**. Power to detect linkage *θ* of level *α* = 0.001 LOD score test by using *N* = 300 doubly affected relative pairs **(B)** and *N* = 300 extreme discordant relative pairs with *QTL* **(D)**.

(11) T=2lgϵ^Nϵ^(1−ϵ^)N(1−ϵ^)ϵ0Nϵ^(1−ϵ0)N(1−ϵ^),

where $\epsilon_{0}$ is the conditional marker *IBD* probabilities under null hypothesis. Thus, the likelihood ratio test statistic *T* asymptotically distributed as *χ*^2^ with 1 d.f. Defining equation (11) as *T*(*N_j_, N*), and assuming level-*α* test with 1 −*β* power, I obtain {*N_j_, N*} for each relative relationship as the critical size of relative pairs sharing allele *IBD* and total required sample size, respectively. One can check easily that *T* is an increasing function of *N_j_* when *N*s are fixed. In other words, for an each *N*, I reject the null hypothesis if the counts of allele *IBD* are greater than *N_j_*. Usually, the LOD score test use more strict criterion than the proportion test does. Here, the total required sample size *N* of the 90% power, level *α* = 0.001 LOD score test power is plotted as a function of the recombination fraction *θ* for both doubly affected relative pairs with the dichotomous trait and extreme discordant relative pairs with *QTL* (Figures 2A,C). In many respects, they behave similarly such that sib pairs have larger power for low *θ*, while grandparent–grandchild pairs have the best power for high *θ* (Figures 2B,D). In general, both critical allele *IBD* sharing size *N_j_* and total relative pair size *N* are increasing as *θ* gets closer to 0.5 or as the power of the test increases.

### 3.3 Mean Test

Since *N* interested sib pair can share either two or one allele(s) *IBD*, I weight *N*_1_ with $\frac{1}{2}$, and define $T_{s-m}=N_{2}+\frac{1}{2}N_{1}$, the Wald test statistics is:

(12) $$W_{s-m}=\sqrt{\frac{2}{N}}\left(2T_{s-m}-N\right).$$

Under the alternative hypothesis of $\theta <\frac{1}{2}$, *W*_*s*−*m*_ is approximately normally distributed with

(13) $$W_{s-m}∼N\left(\sqrt{N_{s-m}}\left(2\epsilon_{2}+\epsilon 1-1\right),8\epsilon_{2}\left(1-\epsilon_{2}\right)\right.\;\left.+\;2\epsilon_{1}\left(1-\epsilon_{1}\right)-8\epsilon_{2}\epsilon_{1}\right).$$

by Central Limit Theorem, where $\epsilon_{2}$ and $\epsilon_{1}$ are conditional marker *IBD* probabilities for sib pair sharing two or one allele(s) *IBD*, respectively. For sib pair, one expects the increased allele sharing under the alternative hypothesis, the level-*α* one-sided mean test is: *W_s–m_* > Z*_α_* for doubly affected sib pairs with the dichotomous trait and *W_s–m_* < −Z*_α_* for extreme discordant sib pairs with *QTL*. Following similar procedure as the proportion test, I obtain the required sib pair sample size *N_s–m_* for level-*α* mean test with power 1−*β* (14):

(14) $$N_{s-m}\;={\left(\frac{\pm \;Z_{\alpha }-\sqrt{2}Z_{1-\beta }\sqrt{4\epsilon_{2}\left(1-\epsilon_{2}\right)+\epsilon_{1}\left(1-\epsilon_{1}\right)-4\epsilon_{2}\epsilon_{1}}}{\sqrt{2}\left(2\epsilon_{2}+\epsilon_{1}-1\right)}\right)}^{2}.$$

**Figure 3:**
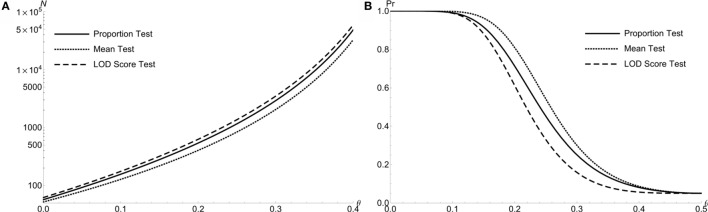
**The power comparison of the proportion test, the LOD test and the mean test for doubly affected Sib Pairs with the dichotomous trait**. **(A)** Required sample size *N* of 3 different test statistics at level *α* = 0.05 with 90% power to detect linkage *θ*. For doubly affected sib pairs. **(B)** Power to detect linkage *θ* of 3 test statistics at level *α* = 0.05 by using *N* = 300 doubly affected sib pairs.

Figure 3A compares the required total sample size *N* of doubly affected sib pair with the dichotomous trait for all three test at *α* level of 0.05 with 90% power: the proportion test (solid line), the mean test (dotted line), and the LOD score test (medium dash). The mean test for sib pair requires the least sample size than other two. For example, the required sample sizes are {157, 128, 176} for the proportion, mean and LOD score tests, respectively, when *θ* = 0.1. Here, the mean test demonstrates the largest test power among all three tests (Figure 3B). The results are similar for extreme discordant sib pairs (figures not shown).

### 3.4 Simulation Study

**Figure 4:**
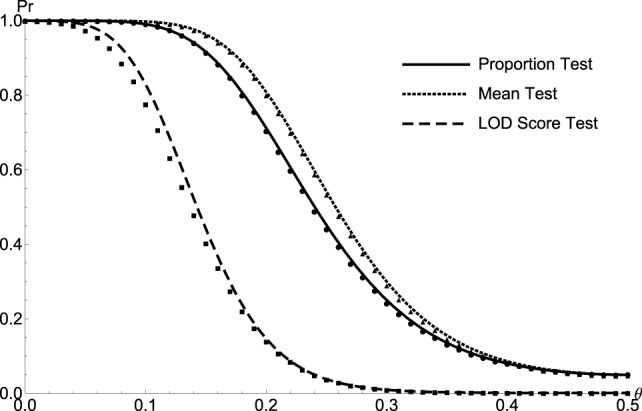
**The comparison between the simulated and theoretical test powers of Sib Pairs**. The simulated/calculated powers for proportion test (solid line/circle), the mean test (dotted line/triangle), and the LOD score test (medium dash/square) are plotted as a function of recombination fraction *θ* for sib pairs.

In this section, I perform the Monte-Carlo simulation procedures to evaluate the power of three statistical tests. The pedigree data consists of 300 replicates of 5 nuclear families. Within each nuclear family, there are two affected individuals with the dichotomous trait representing relative relationship of sibs, grandparent–grandchild, uncle–nephew, half sibs, and first cousin. Since the simulation programs use the parameters set {*p, f*_1_, *f*_2_, *f*_3_}, I take only one reasonable solution set for {*K_P_, V_A_, V_D_*} = {0.1, 0.01, 0.01}, where *p* = 0.7887 is the gene frequency of the normal allele, *f*_1_ = 0.05359, *f*_2_ = 0.1 are the first two penetrance frequencies of homozygous individual of normal alleles, and heterozygous individual, *f*_3_ = 0.7464 is the penetrance frequency of homozygous individual carrying recessive disease alleles. Total 100,000 data set were generated under different hypothesis of *θ*. The test power was then evaluated at putative *α* level of 0.05 for the proportion and mean test statistics, and *α* level of 0.001 for the LOD score test statistic. The simulated empirical powers are consistent with the theoretical calculations for all relative relationships, which serve as a validation of the test statistics, and result of sib pair is shown in Figure 4.

## 4 Discussion

I have demonstrated the *Pr*(*IBD_M_*|*X*) perturbation is closely related to the power to linkage tests. When *V_A_* = 0, the non-zero perturbation of full sib pair is due to the *V_D_* term. However, there are no *V_D_* term in the perturbations of other relative relationships, *i.e*., the perturbations are always zero, whenever *V_A_* hits zero for relative relationship, grandparent–grandchild, uncle–nephew, half sibs, and first cousin. Among all the relative relationships, only the perturbation of grandparent–grandchild shows linear dependence upon the recombination fraction, *θ*, while the remaining perturbations are higher order polynomial functions of *θ*. One also notices that the condition of *θ* = 0 and $V_{A}=\frac{27}{128}$ yields the maximal perturbations for all relative relationships: 0.2394 for full sibs, 0.5438 for first cousin, and equal maximal perturbation of 0.4203 for grandparent–grandchild, uncle–nephew and half sibs. Thus, for the relative relationships, grandparent–grandchild, uncle–nephew, and half sibs, the tests start with equal sample size, *N* = 211 at *θ* = 0 (in Figure 1A) and *N* = 298 at *θ* = 0 (in Figure 2A). This conclusion also holds for extreme discordant relative pair with *QTL*.

There exist programs that could evaluate the type I error rate of the three statistical tests under the null hypothesis of no linkage. The marker genotypes of each relative pair are independently generated by either **SLINK** or **SIMULATE** programs. The **SLINK** program randomly predicts the marker genotypes by calculating their conditional probabilities given the disease phenotypes (23, 24), while the **SIMULATE** program simulates pedigree data by using a crossover formation (CF) process to generate the counts of crossovers and their locations along a chromosome (25). Once the pedigree files have been created by either **SLINK** or **SIMULATE** program, test statistics are calculated through exact counts of relative pairs sharing allele(s) *IBD*. The empirical type I error rates generated by both programs are consistent with the nominal *α* levels (results not shown). However, neither **SLINK** nor **SIMULATE** could track allele segregation unambiguously under the alternative hypothesis. Therefore, I constructed Monte-Carlo simulation directly from Table 5, so that the tests’ power could be evaluated under both null and alternative hypotheses.

Because the counting statistic relies on the number of alleles shared *IBD* in affected relative pairs to detect linkage, informativeness of the marker *IBD* determines the accuracy of linkage analysis. A marker is highly informative for linkage studies, if any individual chosen at random is likely to be heterozygous for that marker. Nonetheless, in almost all applications, the biallelic *IBD* value can not be determined unambiguously, but has to be estimated. Previous work has been shown that increased information of allele shared *IBD* of sib pair can be achieved by analyzing two or more linked marker loci simultaneously (26, 27). In order to recapture the lost information, Kruglyak et al. and Kong and Cox have performed weighting schemes to take account of all pedigree information (28, 29). Buckman and Li combined both alleles *identical by descent* (*IBS*) and *IBD* missing at random (MAR) into the test statistic which has equal power as those in Kong and Cox (30).

The allele-sharing methods, originally designed for application of affected sib pair, are also referred as model-free (no assumption of the distribution) linkage analysis and advantageous over traditional model based methods. Thus, this method does not require specification of the disease model and could be readily applied to either early- or late-onset disease. In practice, samples collected for affected relative pair will likely contain three or more affected relatives, such as siblings, grandparent–grandchild, uncle–nephew, half sib, or first cousin. However, most commonly used methods, restrict the linkage analysis to sib pair only. Thus, a large amount of information contained in the data is discarded. The simple way to achieve larger power is to include all available affected individuals from each relative type. Since the possible selected pairs are no longer independent, several weighting schemes were applied to sib pair (18, 31). The most powerful weighting scheme for various relative pairs are still need to be considered, perhaps their theoretical sample size and power could be calculated.

## Author Contributions

QZ contributed to the research topic, derived model formulation, carried out the numerical simulation, and wrote the manuscript.

## Conflict of Interest Statement

The author declares that the research was conducted in the absence of any commercial or financial relationships that could be construed as a potential conflict of interest.
